# *Wolbachia* strains *w*Mel and *w*AlbB differentially affect *Aedes aegypti* traits related to fecundity

**DOI:** 10.1128/spectrum.00128-24

**Published:** 2024-03-14

**Authors:** Rafael Maciel-de-Freitas, Felix G. Sauer, Konstantin Kliemke, Gabriela A. Garcia, Márcio G. Pavan, Mariana R. David, Jonas Schmidt-Chanasit, Ary Hoffmann, Renke Lühken

**Affiliations:** 1 Bernhard Nocht Institute for Tropical Medicine, Hamburg, Germany; 2 Laboratório de Mosquitos Transmissores de Hematozoários, Instituto Oswaldo Cruz, Fiocruz, Rio de Janeiro, Brazil; 3 Instituto Nacional de Ciência e Tecnologia em Entomologia Médica, Universidade Federal do Rio de Janeiro, Rio de Janeiro, Brazil; 4 Faculty of Mathematics, Informatics and Natural Sciences, University of Hamburg, Hamburg, Germany; 5 Pest and Environmental Adaptation Research Group, School of BioSciences, Bio21 Institute, The University of Melbourne, Melbourne, Australia; CSIR - Institute of Microbial Technology, Chandigarh, India

**Keywords:** *Aedes aegypti*, *Wolbachia*, vectorial capacity, disease transmission, blocking, life history traits

## Abstract

Two *Wolbachia* strains, *w*Mel and *w*AlbB, have been transinfected into *Aedes aegypti* mosquitoes for population replacement with the aim of reducing dengue transmission. Epidemiological data from various endemic sites suggest a pronounced decrease in dengue transmission after implementing this strategy. In this study, we investigated the impact of the *Wolbachia* strains *w*Mel and *w*AlbB on *Ae. aegypti* fitness in a common genetic background. We found that *Ae. aegypti* females infected with the *w*Mel strain exhibited several significant differences compared with those infected with the *w*AlbB strain. Specifically, *w*Mel-infected females laid significantly fewer eggs, ingested a lower amount of blood, had a reduced egg production rate, and exhibited a decreased *Wolbachia* density at a later age compared with mosquitoes infected with the *w*AlbB strain. Conversely, the *w*AlbB strain showed only mild negative effects when compared with *Wolbachia*-uninfected specimens. These differential effects on *Ae. aegypti* fitness following infection with either *w*Mel or *w*AlbB may have important implications for the success of population replacement strategies in invading native *Ae. aegypti* populations in endemic settings. Further research is needed to better understand the underlying mechanisms responsible for these differences in fitness effects and their potential impact on the long-term efficacy of *Wolbachia*-based dengue control programs.

**IMPORTANCE:**

The transmission of arboviruses such as dengue, Zika, and chikungunya is on the rise globally. Among the most promising strategies to reduce arbovirus burden is the release of one out of two strains of *Wolbachia*-infected *Aedes aegypti*: *w*Mel and *w*AlbB. One critical aspect of whether this approach will succeed involves the fitness cost of either *Wolbachia* strains on mosquito life history traits. For instance, we found that *w*Mel-infected *Ae. aegypti* females laid significantly fewer eggs, ingested a lower amount of blood, had a reduced egg production rate, and exhibited a decreased Wolbachia density at a later age compared with mosquitoes infected with the *w*AlbB strain. Conversely, the *w*AlbB strain showed only mild negative effects when compared with *Wolbachia*-uninfected specimens. These differential effects on mosquito fitness following infection with either *w*Mel or *w*AlbB may have important implications for the success of population replacement strategies in invading native *Ae. aegypti* populations.

## INTRODUCTION

Arthropods usually host a wide variety of microorganisms, some of which live in an intimate and long-term biological interaction with their host. The interactions between insects and viruses, bacteria, and fungi can be classified into a gradient ranging from benefits to both parties involving mutualism (1) to antagonistic interactions resulting in parasitism (2, 3), both of which are driven by evolutionary changes in the hosts and the microbes (4, 5). Microorganisms are important modulators of host phenotypes, providing heritable variation acted upon by natural selection (6). Host-parasite interactions represent one of the strongest selection pressures in nature, with considerable impact on the ecology and evolution of the microbes and thus on disease epidemiology (7). These biological interactions in a changing environment form part of a complex ecosystem shaping disease transmission.

Due to epidemiological impacts on human health, it is of paramount importance to understand host-parasite interactions within the context of vector-borne diseases. Among mosquitoes, special interest has been given to *Aedes aegypti*, the primary vector of arboviruses such as dengue (DENV), Zika (ZIKV), and chikungunya (CHIKV). DENV is organized in four distinct serotypes (DENV-1, -2, -3, and -4), with an estimated infection of 390 million people per year globally (8–10). In 2013, ZIKV rapidly disseminated from the Pacific islands to Southern America and became a global public health emergence due to its association with microcephaly in newborns (11). At least two CHIKV lineages have spread over the world (ECSA—the East-Central-South Africa and the Asian lineage), causing severe outbreaks in sites where *Ae. aegypti* was abundant (12, 13).

In the absence of widely applicable, effective vaccines for most arboviruses, mitigation efforts rely on suppressing and maintaining vector populations below a threshold at which outbreaks are unlikely to occur (14). Traditional vector control methods have had limited success in managing arbovirus outbreaks, due to factors such as widespread insecticide resistance in vector populations and the huge effort required in targeting key breeding sites in urban settings in the long term (15–17). Therefore, new strategies to supplement traditional vector control methods need to be developed to manage mosquito-borne diseases. One innovative approach currently undertaken in at least 14 countries is the release of *Ae. aegypti* carrying *Wolbachia*, an endosymbiont naturally present in around 40% of insect species but naturally absent from *Ae. aegypti* (18). *Wolbachia* can be successfully established in *Ae. aegypti* using embryo cytoplasm microinjection (19). So far, two artificially *Wolbachia*-infected strains of *Ae. aegypti* have been released in the field: *w*Mel (transinfected from *Drosophila melanogaster*) and *w*AlbB (transinfected from *Ae. albopictus*). *Wolbachia* strains in *Ae. aegypti* can induce density-dependent blocking of DENV, ZIKV, and CHIKV, which is favored by the high density of this bacterium in salivary glands (20–22). The spread of *Wolbachia* in the mosquito population following release is facilitated by two attributes: maternal transmission to offspring, coupled with cytoplasmic incompatibility, wherein *Wolbachia*-infected males induce sterility in wild females, producing a frequency-dependent fitness advantage for *Wolbachia*-infected females (23, 24). So far, *w*Mel or *w*AlbB strains have been released in countries such as Australia, Brazil, Indonesia, Malaysia, and Vietnam to replace natural populations highly competent to arbovirus by *Wolbachia*-infected specimens with reduced vector competence (25–28).

Although available data point to a reduction in dengue incidence due to *Wolbachia* deployment (28–30), a number of important issues remain unresolved regarding both the short- and long-term interaction between the bacterium and *Ae. aegypti* mosquitoes. For example, *Wolbachia* is known to affect invertebrate fitness and reproduction and generate several physiological changes in mosquitoes (31–34), but so far, there are limited data on how the released strains (*w*Mel and *w*AlbB) affect *Ae. aegypti* reproductive strategies. Trade-offs within an organism’s life history result from allocation of a fixed resource budget among growth, survival, and reproduction (35, 36). For instance, *w*Mel-infected *Ae. aegypti* have a significant decrease in egg hatching, which could likely limit the spread of this strain within a native *Ae. aegypti* population (26, 37). There remains a gap in understanding whether the two *Wolbachia* strains currently deployed can impact *Ae. aegypti* reproductive tactics, particularly on genetic backgrounds from areas where the strategy is being deployed. Therefore, this study aimed to analyze the effect of *Wolbachia* infection on *Ae. aegypti* with a Brazilian genetic background for different fitness-related mosquito traits, including wing length, wing shape, blood meal size, and fecundity.

## MATERIALS AND METHODS

### Wild mosquitoes and backcrossing

We used three *Ae. aegypti* strains in this study. To represent a *Wolbachia*-uninfected field population, we sampled eggs in the neighborhood of Urca (22°56′44″S, 43°09′42″W). Previous reports highlighted that insecticide resistance in *Wolbachia*-carrying mosquitoes (*w*Mel strain) plays a role in the bacterium being able to invade into wild *Ae. aegypti* populations. Mosquitoes from Rio de Janeiro are resistant to pyrethroids, and insecticide resistance is a widely spread phenomenon in Brazil (16, 26, 38–41), highlighting the importance of controlling the genetic background. Urca is located 12 km away from the southern geographic limit of the area in which *Wolbachia* has been released in Rio de Janeiro. Thus, *Wolbachia-*infected individuals are not expected to be present in this neighborhood (42). We sampled eggs in Urca using 60 ovitraps with wooden paddles replaced weekly for ~2 months until we obtained a minimum of 10,000 eggs to capture local genetic diversity. Eggs were hatched in plastic containers with 3 L of tap water and yeast in the week following their collection in the field. Larvae were fed daily with fish food (4.5 mg) until the pupae stage. Pupae were transferred to 30 × 30 × 30 cm cages (BugDorms, Taichung, Taiwan) to allow adult emergence and mating. Two cages with at least 500 *Ae. aegypti* females in each were established, with females blood fed twice a week to obtain eggs.

The *w*Mel and *w*AlbB strains were originally created through microinjection into *Ae. aegypti* eggs of an Australian background (43, 44). By backcrossing *Ae. aegypti* females from the lines to field-collected males for five consecutive generations, we ensured that the material used in experiments has a similar genetic background to those of field mosquitoes, allowing us to isolate phenotypic effects specifically associated with *Wolbachia*. After colonies were closed, i.e., when the backcrossing was concluded, genetic variation in the colonies of *Wolbachia* lines and genetic similarity to the Urca population was maintained by ensuring that around 50% of males were from the *Wolbachia*-uninfected Urca-derived colony every two generations (26). By doing so, we refreshed the genetic background of infected colonies, ensuring that mosquitoes from the three strains have a similar genetic background. All colonies were maintained with around 500 females each in an insectary at 27 ± 2°C, 14:10 light:dark photoperiod, >60% relative humidity. For the experiments reported herein, we used eggs derived from two independent cages of each colony, i.e., in duplicate. The egg hatching and larval rearing methods were the same as stated above.

### Blood feeding

Adult mosquitoes were supplied with 8% sucrose diet *ad libitum* until 24 h before blood feeding. A total of six cages was used in this experiment, two for each mosquito strain, with males and females mixed from the two cages. Each cage had approximately 250 *Ae. aegypti* females. A blood meal was offered for one cage of each strain when *Ae. aegypti* females were approximately 1 week old (7–8 days after emergence). Females in the second cage of a strain received their first blood meal when they were 3 weeks old (21–23 days after emergence). Given what is known about *Ae. aegypti* reproductive biology, we expected that after 1 week, all females would have been inseminated. Blood feeding was carried out with expired human blood group 0 purchased as blood bags from local blood banks. The blood donor’s private information is unknown, i.e., ethical approval is not required. The blood was offered with a Hemotek Membrane Feeding System (Hemotek Ltd., Blackburn, UK), and females were allowed to feed for half an hour. Visually completely engorged females were individualized in 50-mL plastic vials containing moistened cotton overlaid with filter paper as oviposition substrate on the bottom and a sugar solution 8% *ad libitum* on the top. Tubes were covered on the top with mosquito netting.

### Blood meal size and egg production

Females remained inside the vials for 1 week after blood feeding in the same insectary conditions as the colonies described above. After this period, mosquitoes were killed by freezing and stored for wing removal and *Wolbachia* quantification. Eggs laid on the filter paper were counted with the aid of a stereomicroscope at 10× magnification (Leica M205 C, Leica Microsystems, Wetzlar, Germany), recorded, and later discarded for estimating the blood meal size. We measured the blood meal size by quantifying the hematin from mosquito feces on the filter paper. The filter papers were added to a 2-mL cuvette (Sarstedt, Nümbrecht, Germany) containing 1 mL of a 1% lithium carbonate (Sigma Aldrich, Burlington, USA) solution to dilute the feces. A standard line was prepared by diluting known amounts of blood (0, 0.8, 1.6, 2.4, and 3.2 µL) and measuring the corresponding hematin in a spectrophotometer (Thermo BioMate 3) with an absorbance at 387 nM (45–48). The standard line had an *R*^2^ of 0.9832 in a linear regression. By dividing the number of eggs laid per female by the amount of blood ingested (transformed using the standard), we were able to evaluate the individual efficiency of egg production with a known amount of blood, a ratio expressed as eggs/ug.

### Wing shape and length

The right and left wings of each specimen were removed and mounted under a cover slip (15 × 15 mm) with Euparal (Carl Roth, Karlsruhe, Germany). Pictures of each wing were taken with a stereomicroscope (as above) at 20× magnification. Fiji (49), a bioscience package of ImageJ (50), was used to digitize 18 landmarks. The landmark selection was in accordance with other studies using geometric morphometrics to analyze the wing shape of mosquitoes (51–53). The wing length was measured as the distance from the axillary incision to the apical margin excluding the fringe (54).

### *Wolbachia* quantification

*Wolbachia* was quantified on the whole body of each specimen without the wings. *Wolbachia* DNA was extracted with a DNeasy Blood & Tissue Kits (Qiagen, Hilden, Germany), following the manufacturer’s instructions and previous experiments (44, 55, 56). Detection of *Wolbachia w*Mel strain was based on amplification of the WD0513 gene. The following primers were used to amplify a fragment of 110 bp: TM513-F: 5′-CAAATTGCTCTTGTCCTGTGG-3′ and TM513-R: 5′-GGGTGTTAAGCAGAGTTACGG-3′, and the probe 5′-FAM-TGAAATGGAAAAATTGGCGAGGTGTAGG-3BHQ1-3′. In the same reaction, a ribosomal gene from *Ae. aegypti* (RPS17) with the length of 68 bp was amplified with the two primers: RPS17-F: 5′- TCCGTGGTATCTCCATCAAGCT-3′ and RPS-R: 5′- CACTTCCGGCACGTAGTTGTC-3′ and the probe RPS17: 5′-HEX-CAGGAGGAGGAACGTGAGCGCAG-BHQ1-3′. The amplification was carried out on a Qiagen Rotor-Gene Q using Taqman Universal PCR Master Mix (Thermo Scientific, Waltham, USA) following the manufacturer’s instructions. The relative quantification of *w*Mel strain relative quantification was performed according the procedure described elsewhere (55). The *Wolbachia w*AlbB strain was detected by high-resolution melting polymerase chain reaction (qPCR-HRM) (56) with 1:10 diluted DNA using the following *w*AlbB1-specifc primers: *w*AlbB1-F (5′-CCTTACCTCCTGCACAACAA-3′) and *w*AlbB1-R (5′-GGATTGTCCAGTGGCCTTA-3′), as well as universal mosquito primers: mRpS6_F (5′-AGTTGAACGTATCGTTTCCCGCTAC-3′) and mRpS6*_*R (5′-GAAGTGACGCAGCTTGTGGTCGTCC-3′), which target the conserved region of the RpS6 gene, and *Ae. aegypti* primers aRpS6*-*F (5′-ATCAAGAAGCGCCGTGTCG-3′) and aRpS6-R (5′-CAGGTGCAGGATCTTCATGTATTCG-3′), which target the *Ae. aegypti-*specific polymorphisms within RpS6 and do not amplify *Ae. albopictus*. Reactions were run as 384-well plates in a LightCycler 480 II (Roche, Basel, Switzerland). qPCR-HRM was performed following the same cycling conditions as described elsewhere (28). Samples were considered positive for *Wolbachia* when the Tm for the amplicon produced by the *Ae. aegypti* primers was at least 84°C and the Tm for the *Wolbachia*-primer amplicon was around 80°C. Differences between the Crossing points (Cp) of the *Wolbachia* and *Ae. aegypti* markers were transformed by 2^n^ to obtain approximate estimates of *Wolbachia* density. Negative and positive controls were used in all reactions as non-infected strain and lab colony samples that tested positive in previous assays (37, 57).

### Data analysis

The data set comprised six variables: number of eggs (count data); wing length, blood meal size, and *Wolbachia* density (continuous variables); age (1st or 3rd week); and strain (*Wolbachia*-uninfected, *w*Mel, and *w*AlbB) as categorical variables. Variables were treated as response or explanatory variables depending on the research hypothesis tested. Depending on the normality of the response variable, comparisons within categories of interest were performed using one-way analysis of variance (ANOVA), followed by Tukey-Kramer post test, or Kruskal Wallis (KW) followed by pairwise Wilcoxon test when the null hypothesis of equal means or medians across treatments was rejected. In both cases, the Bonferroni-adjusted significance level was adopted. To verify the effects of explanatory variables on the response variables with count or continuous data, we performed Generalized Linear Model (GLM) analyses and followed a model selection approach. The GLMs were developed as follows. First, we tested the normality and homoscedasticity of variances within the count and continuous variables using the Shapiro-Wilk test. The family distribution of the response variable was assessed through the Cullen and Fray graph, and dispersion was tested using the “RT4Bio” and “fitdistrplus” packages [v1.1; (58, 59)]. The effects of *Wolbachia* density (for the *w*Mel and *w*AlbB groups), blood meal size, wing length, female age, and strain on the number of eggs laid per *Ae. aegypti* female were analyzed with GLM with a negative binomial distribution. This distribution was preferred over the traditionally used Poisson distribution, because data exhibited overdispersion (i.e., variance was larger than the mean), confirmed by Pearson’s chi-squared test and dispersion statistic > 1. Blood meal size and *Wolbachia* density as response variable were analyzed by GLMs with a Gaussian distribution. The variance inflation factor of the models were accessed using the “vcd” package [v1.4–11 (60)]. Variables were removed when multicollinearity was detected, i.e., when GVIF > 5. The most informative and parsimonious model was selected through delta Akaike’s Information Criteria scores corrected for small sample sizes (ΔAICc). Akaike weights were used to assess the uncertainty of model selection, which quantifies the probability that the model is the best among all models built for that response variable (61, 62). For each set of models created for a given response variable, we selected the best model(s) for interpretation of its parameters if ΔAICc was less than 2.0 (61, 62). Multicollinearity was checked again in the best model. Finally, the assumptions of the best model were examined by checking heteroscedasticity, residual dispersion, and the presence of outliers with the package “DHARMa” [v.0.4.6 (63)]. All analyses were done in the R environment (64).

**Fig 1:**
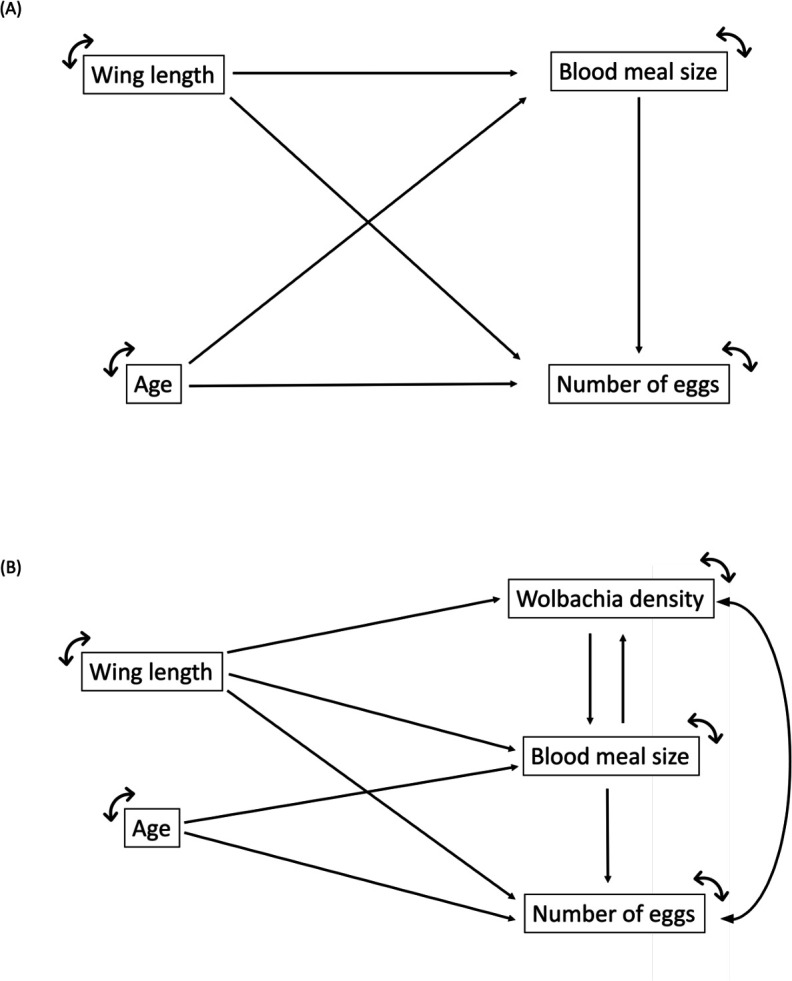
Hypothesized path diagram considering *Ae. aegypti* age, wing length, blood meal size, and the number of eggs. (**A**) The *Wolbachia*-uninfected field strain; (**B**) the two strains with *Wolbachia* (*w*Mel and *w*AlbB strains). The notations of the path diagram are as follows: age is an exogenous variable, whereas wing length, blood meal size, *Wolbachia* density, and the number of eggs are endogenous variables. The arrows represent the directional relationship, and circled double arrows represent the variance and the residual errors of exogenous and endogenous variables, respectively.

Path analysis or Structural Equation Modeling (SEM) is a multivariate regression technique that allows the direction and magnitude of each of the direct effects (path coefficients) on the response variable (65) to be obtained. The main purpose of SEM is to confirm an agreement between specific causal hypotheses and empirical data, which is assessed through a goodness-of-fit statistic between the observed and expected correlations (65, 66). We established a causal relation model to assess whether *Ae. aegypti* age, wing length, blood meal size, and *Wolbachia* density (except for field strain) affect the number of eggs laid by female mosquitoes (Fig. 1). A principal component analysis (PCA) using the package “psych” in R (67) showed the data differ among the three strains, with no overlapping clusters between *Wolbachia*-infected strains and the *Wolbachia*-uninfected strain (Fig. S1). Therefore, we built a path diagram based on the SEM approach for each strain. Our global model used mosquito age as an exogenous variable and wing length, blood meal size, *Wolbachia* density (when possible), and the number of eggs as endogenous variables. Alternative path diagrams were tested by comparing coefficients of non-determination from reduced models to those of the full model using the same model selection approach described above. Total-effect coefficients (the sum of the direct and indirect effects of one variable on another) were calculated for each of the endogenous variables in the path diagram. We assessed the model fit of the selected path diagram for each strain using the following model fit index: chi-square test (χ^2^), Comparative Fit Index (CFI), Tucker-Lewis Index (TLI), Root Mean Square Error of Approximation (RMSEA), and the Standardized Root Mean Square Residual (SRMSR). All SEM analyses were done in R with the “lavaan” package [v 0.6–15 (64, 68)].

The two-dimensional landmark coordinates of the 18 vein crosses were used to analyze the wing shape of the mosquitoes. The landmark coordinates were aligned by a Generalized Procrustes Analysis using the R package geomorph (version 4.0.1) (69). The wing shape variation between specimens was visualized with a PCA. The differences in the wing shape between the three strains (Urca, *w*Mel, and *w*AlbB) were statistically compared by a Procrustes ANOVA using the proc.D.lm function (with 1,000 permutations). In addition, we calculated the strain-specific morphological disparity with the “morphol.disparity” function. This function calculates the Procrustes variance for the three different strains using the residuals of a linear model and can, thus, provide information about morphological diversity within the strains. The statistical significance of the calculated Procrustes variance between the three strains was checked pairwise using a randomized permutation test with 499 iterations. All wing shape analyses were conducted separately for the right and the left wing to check the consistency of the results and to avoid duplicated measurements per specimen in the same analysis (70).

## RESULTS

Only three mosquitoes died over the period that insects were monitored. Thus, we monitored the blood meal size, wing length, and number of eggs in a total of 417 *Ae. aegypti* females, 140 from the *Wolbachia*-free strain, 139 from *w*Mel, and 138 from *w*AlbB. The three deaths were recorded in mosquitoes that were blood fed in their 3rd week post emergence. All three strains had 75 *Ae. aegypti* females blood feeding in their 1st week, whereas in the 3rd week, we had 65 insects from the *Wolbachia*-free strain, 64 from *w*Mel, and 63 from *w*AlbB.

### Number of eggs

**Fig 2:**
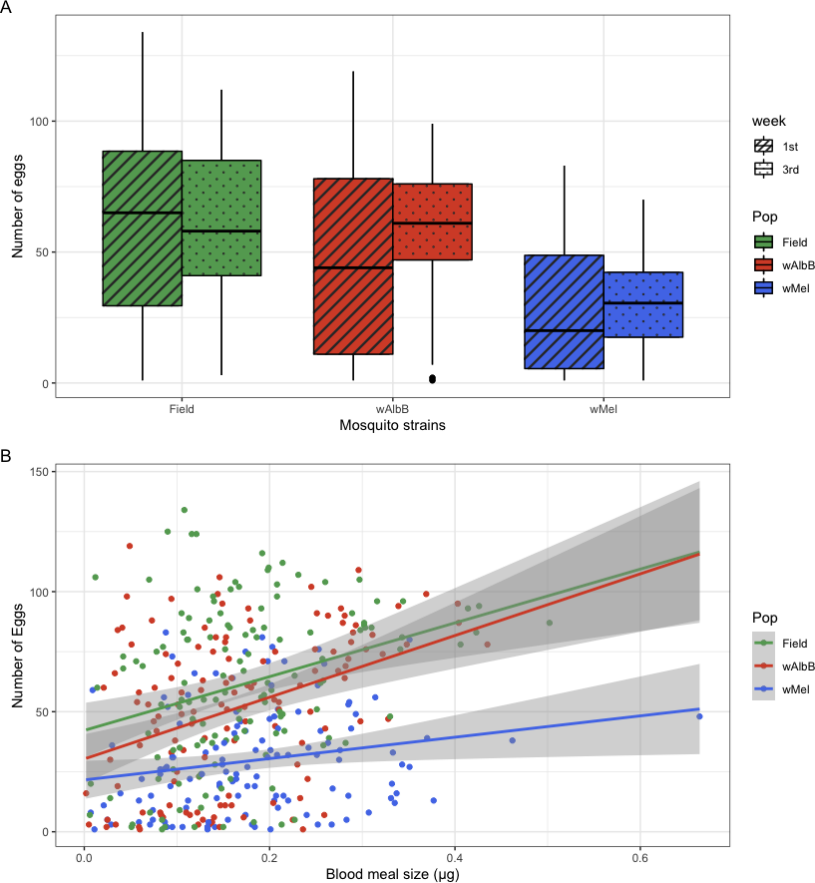
Fecundity of *Aedes aegypti* females from the three tested strains and its association with (**A**) age in weeks and (**B**) blood meal size. The shaded area in **B** represents the 95% confidence level interval for predictions from a linear model.

From the 417 *Ae. aegypti* females, 47 (11.2%) females had not laid any eggs 1 week after blood feeding (8 from the field, 21 from *w*Mel, and 18 from *w*AlbB strain). The maximum number of eggs laid per female was 134. The number of eggs laid by *Ae. aegypti* females varied significantly among strains (KW: χ^2^: 65.56, df = 2, *P* < 0.001), but not with age (KW: χ^2^: 2.88, df = 1, *P* = 0.089) (Fig. 2A), with *Ae. aegypti* females from *w*Mel laying significantly fewer eggs than the field *Wolbachia*-uninfected strain (*P* < 0.001). There was no detectable difference between the number of eggs laid by *w*AlbB and the field strain (*P* = 0.21).

When we used a GLM with a negative binomial distribution to examine various effects on the number of eggs laid by *Ae. aegypti* females, both infected strains had lower fecundity than the *Wolbachia*-uninfected field strain (Table 1). The blood meal size was positively associated with the number of eggs laid (Table 1). However, the strength of the association was not the same among the three strains, with *w*AlbB mosquitoes presenting a trend similar to females from the uninfected strain and *w*Mel mosquitoes showing a weaker association than for the other two strains (Fig. 2B).

**TABLE 1:** Results of the Generalized Linear Model (negative binomial) of the number of eggs laid by *Aedes aegypti* females from three different strains

Term	Estimate	SE	*z*-value	*P* value^*a*^
Strain (*w*AlbB)	−0.225	0.0968	2.946	0.0601
Strain (*w*Mel)	−0.715	0.0956	−2.325	**< 0.001**
Age (3rd)	−0.129	0.0879	−7.484	0.1403
Blood meal size	1.951	0.4699	−1.475	**< 0.001**
Wing length	0.695	0.2417	2.877	**0.0040**

^*a*^ *P* values under 0.05 are bold.

### Blood meal size

**Fig 3:**
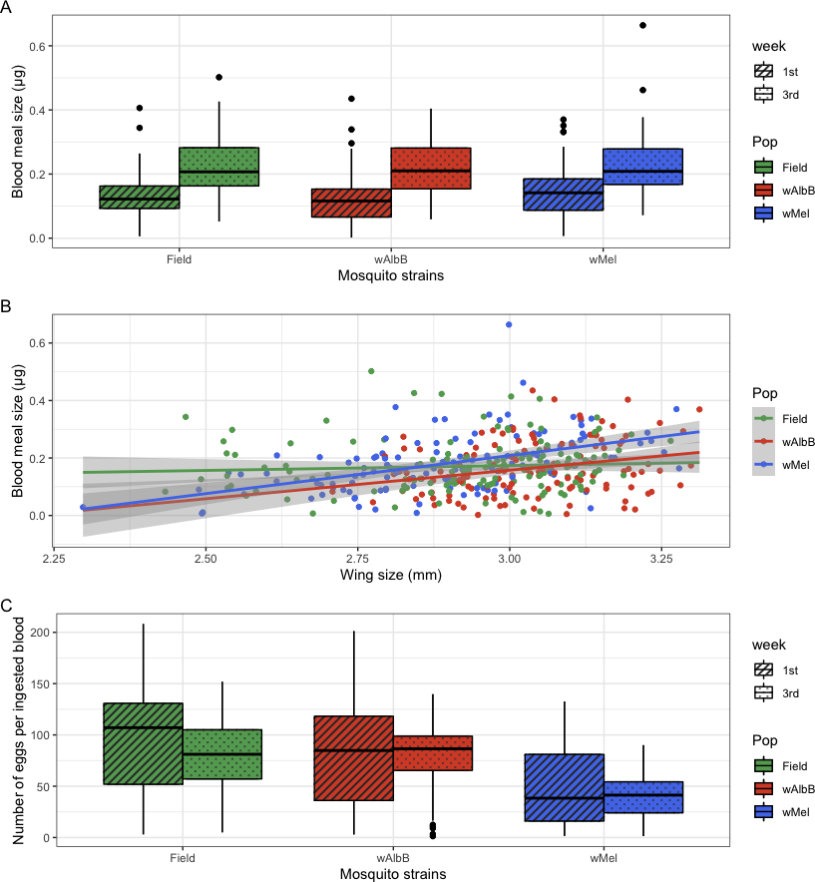
The effects of explanatory variables on the blood meal size of *Aedes aegypti* mosquitoes from three different populations. (**A**) Blood meal size variation according to mosquito strain, (**B**) effects of wing length on the amount of blood ingested by *Aedes aegypti* females, (**C**) egg production based on the amount of blood meal size by *Aedes aegypti* from a *Wolbachia*-uninfected field population, *w*AlbB, and *w*Mel strains, expressed as egg/μg. The shaded area in **B** represents the 95% confidence level interval for predictions from a linear model.

We estimated the blood meal size by quantifying the hematin present on the filter paper. *Aedes aegypti* females from the three strains ingested more blood when fed in the 3rd than in the 1st week (ANOVA, *F*_1,392_ = 106.6, *P* < 0.001) (Fig. 3A). However, the amount of blood ingested did not vary according to the strain (ANOVA, *F*_1,391_ = 1.169, *P* = 0.312).

The best Gaussian GLM considering tblood meal size as the response variable involved no effect of the mosquito strain on the amount of blood ingested by *Ae. aegypti* females. The wing length of mosquitoes was positively correlated with blood meal size (Table 2), although the association varied among strains (Fig. 3B). It is worth noting that the *Wolbachia*-free strain ingested similar amounts of blood regardless of wing length, whereas *Wolbachia*-infected groups behaved similarly, with *w*Mel and *w*AlbB females ingesting more blood when they were bigger. However, the egg production rate significantly varied among strains (KW: χ^2^: 73.71, df = 2, *P* < 0.001), with *Ae. aegypti* infected by the *w*Mel strain producing less egg per blood ingested when compared with *w*AlbB and the *Wolbachia*-uninfected strain (*P* < 0.001 for both paired comparison). The *Wolbachia*-uninfected and *w*AlbB strains had similar egg production (*P* = 0.12). When combined with fecundity and blood meal size variation within age and mosquito strain, the results show that mosquitoes had similar fecundity over ages tested but ingested more blood in the 3rd week, with an overall loss in egg production relative to the amount of blood ingested (Fig. 3C). These loss effects were stronger in the *w*Mel females.

**TABLE 2:** Results of the Generalized Linear Model (Gaussian) of the blood meal size of *Aedes aegypti* females from three different strains

Term	Estimate	SE	*t*-value	*P* value^*a*^
Strain (*w*AlbB)	0.6295	0.3844	1.637	0.1023
Strain (*w*Mel)	−0.0552	0.2857	−0.193	0.8468
Age (3rd)	0.2931	0.1459	2.009	**0.0452**
Wing	0.2087	0.0794	2.628	**0.0089**
Strain(*w*AlbB)*wing	−0.2142	0.1284	−1.668	0.0961
Strain(*w*Mel)*wing	0.0384	0.0938	0.391	0.6960
Age(3rd)*wing	−0.0913	0.0499	−1.828	0.0683

^*a*^ *P* values under 0.05 are bold.

### *Wolbachia* density

**Fig 4:**
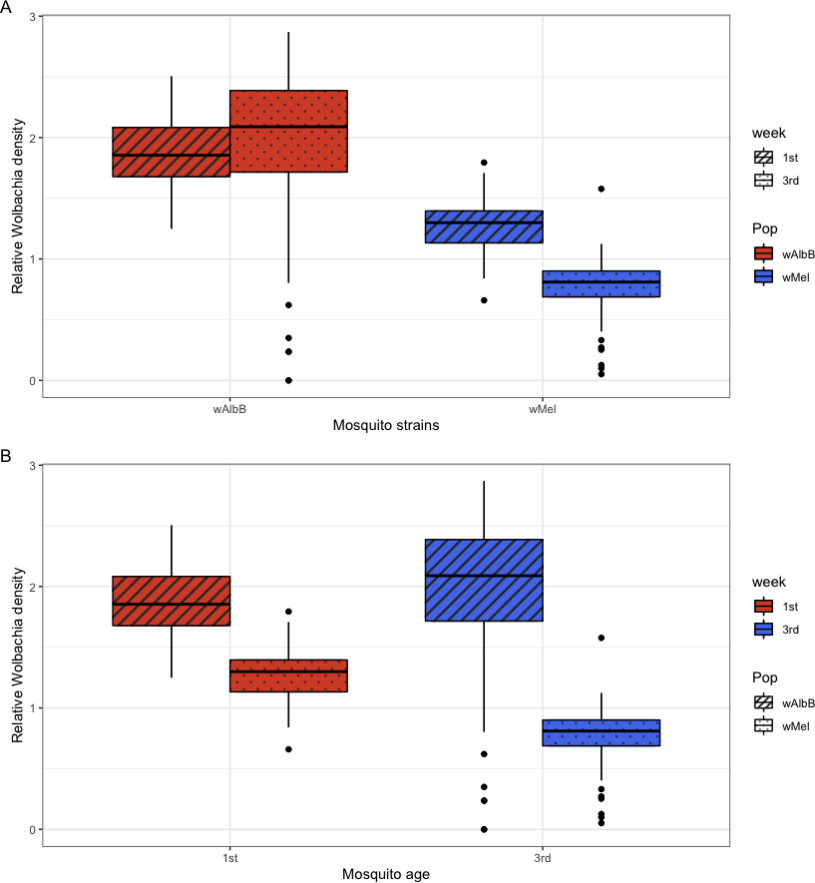
The effects of explanatory variables on *Wolbachia* density considering (**A**) mosquito populations and (**B**) mosquito age.

The *Wolbachia* density was quantified through Reverse transcription-quantitative polymerase chain reaction (RT-qPCR), and *w*AlbB was present in higher densities in *Ae. aegypti* mosquitoes than *w*Mel (ANOVA, *F*_1,276_ = 251.8, *P* < 0.001) (Fig. 4A). At the later age tested, the density of *w*AlbB increased, but for *w*Mel, it decreased (ANOVA, *F*_1,276_ = 8.938, *P* = 0.003) (Fig. 4B). The best model considering *Wolbachia* density as the response variable included an influence of mosquito strain, age, and an interaction between these two independent variables (Table 3).

**TABLE 3:** Results of the Generalized Linear Model (Gaussian) of the *Wolbachia* density of *Aedes aegypti* females from two different strains (*w*Mel and wAlbB)

Term	Estimate	SE	*t*-value	*P* value^*a*^
Strain (*w*Mel)	−4.5889	2.0129	−2.280	**0.0234**
Age (3rd)	0.1437	0.0826	1.739	0.0832
Wing length	−1.1810	0.5569	−2.121	**0.0349**
Blood meal size	−19.471	9.5248	−2.044	**0.0419**
Strain(*w*Mel)*age (3rd)	−0.6838	0.1161	−5.890	**< 0.001**
Strain(*w*Mel)*wing	1.3145	0.6765	1.943	0.0531
Strain(*w*Mel)*blood meal size	19.276	11.3378	1.700	0.0903
wing*blood meal size	6.3156	3.0957	2.040	**0.0424**
Strain(*w*Mel)*wing*blood meal size	−6.1904	3.7341	−1.658	0.0986

^*a*^ *P* values under 0.05 are bold.

### Wing size and shape

**Fig 5:**
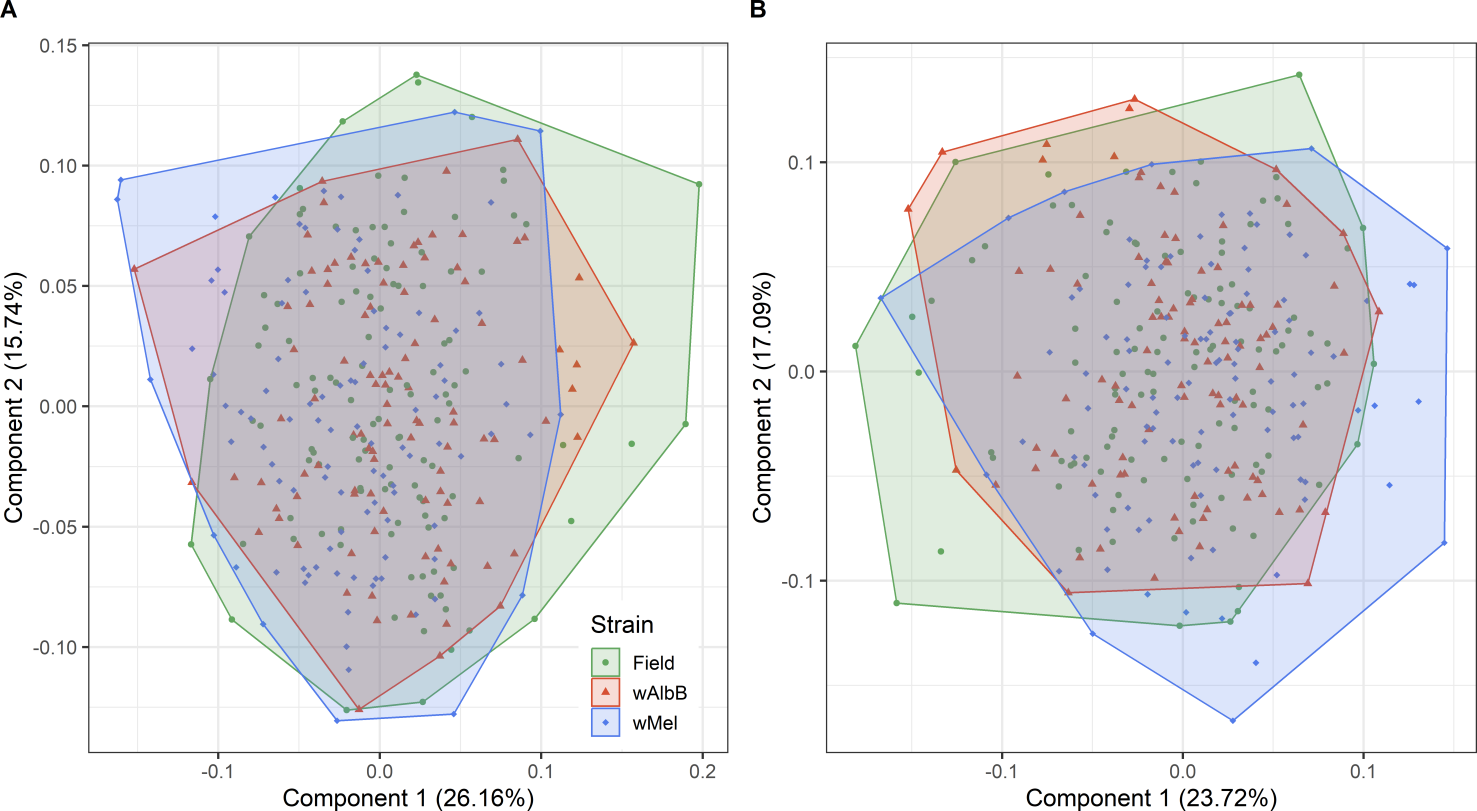
Principal component analysis showing the wing shape variation of the specimens in the left wings (**A**) and right wings (**B**). Green points indicate the specimens from the field population. Red triangles indicate the *w*AlbB-infected population, and blue squares indicate the *w*Mel-infected population.

The wing size varied among strains (KW: χ^2^: 38.65, df = 2, *P* < 0.001), with *w*AlbB bigger than *w*Mel (*P* < 0.001) and the *Wolbachia*-uninfected strain (*P* < 0.001). A marginally non-significant difference was observed between the wing length of *w*Mel and *Wolbachia*-uninfected mosquitoes (*P* = 0.053). The shape of the left wings did not differ significantly between the three strains (ANOVA, *F*_2,316_ = 1.5171, *R*^2^ = 0.0095, *P* = 0.078). In contrast, a small but significant difference was observed for the right wings of the three strains (ANOVA, *F*_2,319_ = 5.358, *R*^2^ = 0.0325, *P* < 0.001) (Fig. 5). The Procrustes variance of the right wings was 0.00125 for the *Wolbachia*-uninfected strain, 0.00123 for the *w*AlbB-infected strain, and 0.00126 for the *w*Mel-infected strain. For the left wings, the Procrustes variance was 0.00122 for the field strain, 0.00119 for the *w*AlbB-infected strain, and 0.00119 for the *w*Mel-infected strain. For both wing sides, pairwise comparisons of the Procrustes variance between the three strains did not reveal statistically significant differences, indicating a similar morphological diversity in the wings of the three strains (Fig. 5).

### Path analysis

**Fig 6:**
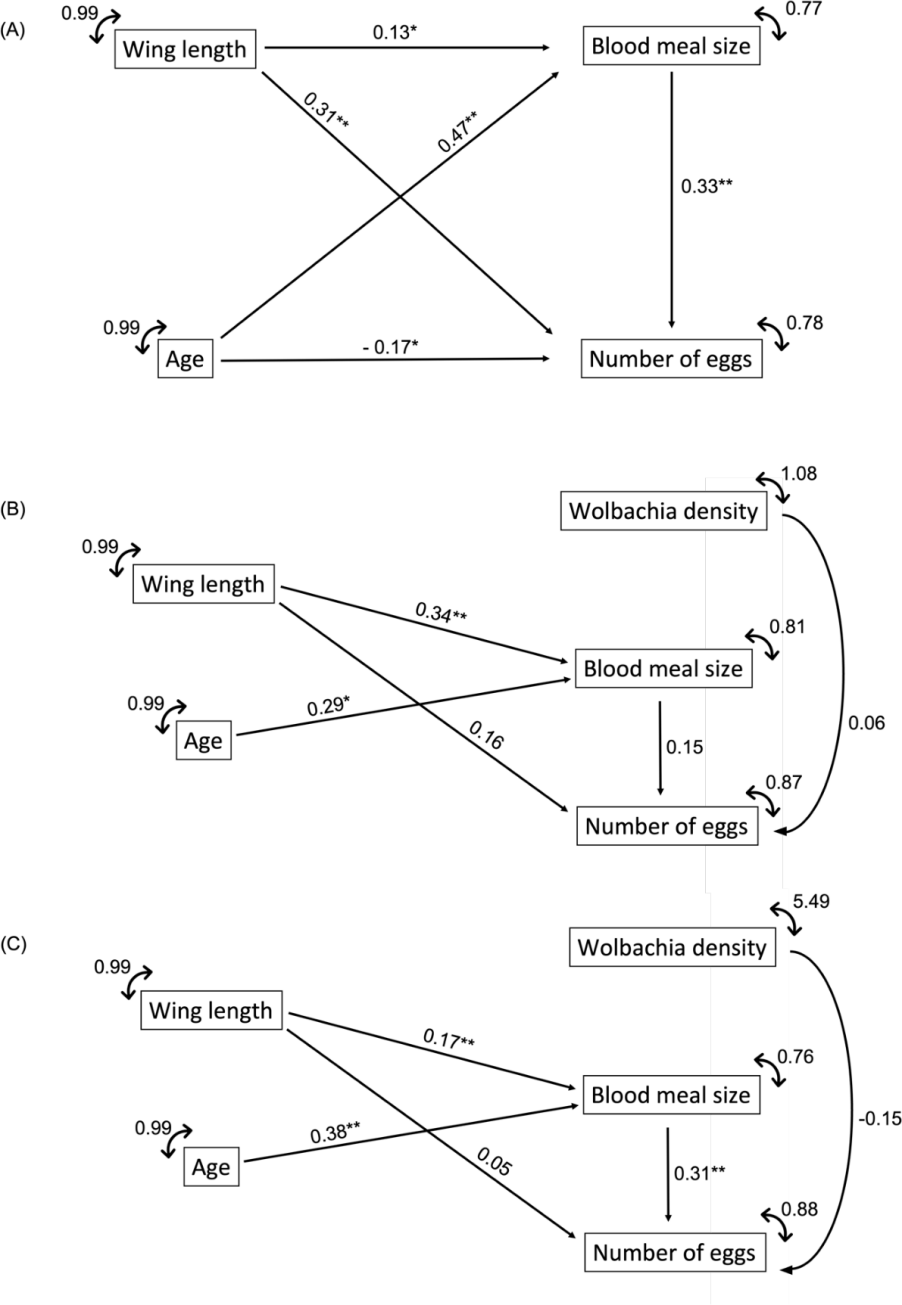
Best model path diagrams for (**A**) *Wolbachia*-uninfected field population, (**B**) *w*AlbB, and (**C**) *w*Mel. Numbers over the arrows are the path coefficients and their statistical significance is indicated as follows: *P < 0.05 and **P < 0.01.

The full model for each strain was classified as overidentified, i.e., with degrees of freedom ≥ 1 (Fig. S2). However, the path diagram of the best model differed for each strain. The best model for the *Wolbachia*-uninfected field strain was the full model, whereas for *w*Mel and *w*AlbB, the best models were reduced versions of the full model, suggesting that metabolic pathways of egg production might be indirectly affected by *Wolbachia* presence (Fig. 6; Table 4). When we identified the best path models, some of the variables included in the full model were omitted for *w*Mel and *w*AlbB. Fecundity in *Wolbachia*-uninfected mosquitoes was positively correlated with blood meal size and wing length but decreased with *Ae. aegypti* at an older age. By comparing the path diagrams of *w*Mel and *w*AlbB mosquitoes, we observed a positive statistically significant effect of wing length and age on blood meal size for both strains but only in *wMel* did the blood meal size significantly affect fecundity. Unlike for uninfected specimens, females carrying either *w*Mel or *w*AlbB showed a non-significant effect of wing size on fecundity. Regarding the presence of *Wolbachia*, the most evident difference between *w*Mel and *w*AlbB was their effect on fecundity: a positive relation regarding *w*AlbB and a negative relationship for *w*Mel. On the other hand, in both path diagrams, the effects of wing length and blood meal size on *Wolbachia* density was removed to increase model fit.

**TABLE 4:** Model fit of full (initial model) and the simplified best path model determining the number of eggs of *Aedes aegypti* females carrying one of the two *Wolbachia* strains (*w*Mel or *w*AlbB) or in the absence of this bacterium^*a*^

Population	Structural equation model	df	χ^2^	CFI	TLI	RMSEA	SRMSR	AIC	ΔAIC
Field (full = best model)	EGG ~ AGE + WNG + BLD, BLD ~ AGE + WNG, AGE ~~ AGE, WNG ~~ WNG, BLD ~~ BLD, EGG ~~ EGG	1	0.143	0.981	0.88	0.093	0.048	1,446.97	-
*w*Mel (full model)	EGG ~ AGE + WNG + BLD + WB, BLD ~ AGE + WNG + WB, WB ~ WNG + BLD, AGE ~~ AGE, BLD ~~ BLD, EGG ~~ EGG, WB ~~ WB	1	<0.001	0.877	0.23	0.395	0.144	1,530.97	-
*w*Mel (best model)	EGG ~ WNG + BLD + WB, BLD ~ AGE + WNG, BLD ~~ BLD, EGG ~~ EGG	2	0.065	0.922	0.726	0.122	0.034	624.57	906.4
*w*AlbB (full model)	EGG ~ AGE + WNG + BLD + WB, BLD ~ AGE + WNG + WB, WB ~ WNG + BLD, AGE ~~ AGE, BLD ~~ BLD, EGG ~~ EGG, WB ~~ WB	1	<0.001	0.669	0.238	0.399	0.112	1,636.52	-
*w*AlbB (best model)	EGG ~ WNG + BLD + WB, BLD ~ AGE + WNG, BLD ~~ BLD, EGG ~~ EGG	2	0.494	0.988	0.954	<0.001	0.024	632.06	1,004.46

^*a*^ EGG, number of eggs; AGE, age of mosquito when blood fed; WNG, wing length; BLD, blood meal size; WB, Wolbachia density; χ^2^, chi-square; CFI, Comparative Fit Index; TLI, Tucker-Lewis Index; RMSEA, Root Mean Square Error of Approximation; SRMSR, Standardized Root Mean Square Residual; AIC, Akaike Information Criteria; DAIC, difference in AIC between the full and best models.

## DISCUSSION

The use of *Wolbachia* to mitigate arbovirus transmission is on the rise in the last few years, with approximately 15 countries conducting release interventions simultaneously. *Wolbachia* population replacement and suppression are influenced by the *Wolbachia* strain considered, mosquito host factors, and environmental variables (24, 71–73). Nowadays, there are two *Wolbachia* strains transinfected into *Ae. aegypti* mosquitoes that are used for population replacement, and for both strains, data suggest a significant reduction in dengue transmission (28, 30, 74). In general, *Wolbachia* infection in *Ae. aegypti* causes a decrease in host fecundity and egg hatching, likely affecting the long-term *Wolbachia* stability under some specific field conditions, particularly those involving high temperatures (37, 75–77). Herein, we adopted a causal relation model followed by structural equation models to determine a path diagram determining how mosquito age, wing length, blood meal size, and *Wolbachia* density affects *Ae. aegypti* fecundity. We observed different path diagrams in the presence of *w*Mel and *w*AlbB, which could potentially affect their success in invading native *Ae. aegypti* populations in endemic settings (26, 78, 79).

By testing a substantial sample size of three strains of *Ae. aegypti* (*Wolbachia*-uninfected *Ae. aegypti*, *w*Mel-infected mosquitoes, and *w*AlbB-infected mosquitoes) in a Brazilian genetic background, we compared the impact of both *Wolbachia* strains on mosquito life history traits. We observed that *w*Mel-infected mosquitoes laid significantly fewer eggs than control mosquitoes, whereas *w*AlbB-infected *Ae. aegypti* had a similar fecundity with control insects. There is evidence in the literature for the detrimental effects of both *Wolbachia* strains on *Ae. aegypti* fitness on host fecundity and fertility, although these effects are not always consistently observed (26, 34, 37, 75, 80, 81). The presence of *Wolbachia* may alter metabolic and physiological processes of *Ae. aegypti* leading to a trade-off impacting mosquito fecundity. For instance, the presence of *w*Mel regulates proteins involved in reactive oxygen species production and regulates humoral immune response and antioxidant production in the ovaries and salivary glands of *Ae. aegypti* females (32, 33). On the other hand, we have not seen any effect of age on fecundity, although *Ae. aegypti* mosquitoes have a trend of reducing the number of eggs laid when aging (46, 82–84). One factor that could explain the lack of aging on the number of eggs laid in our experiment is based on the fact mosquitoes only received one single blood meal and were killed 1 week after for egg counting. Many adult mosquito females will take multiple blood meals during their lifespan, resulting in regular exposure to toxins and blood-meal induced oxidative stress through the secretion of proteolytic enzymes and peritrophic matrix components from midgut epithelial cells, uptake of amino acids, oligopeptides, and lipids through membrane-bound transporter proteins (85–87). Thus, blood feeding-induced mortality in mosquitoes is well characterized, and these mortality/feeding effects need to be considered when testing mosquito lifespan impacts.

Blood meal size, or the amount of blood taken in by female mosquitoes, is believed to regulate several aspects of their biology including host-seeking behavior and fecundity. An intriguing pattern observed for all the three strains is that *Ae. aegypti* females took in larger blood meals when they were 21–23 days old. The effects of aging on *Ae. aegypti* blood meal size have been explored in at least two studies, and in both of them, the amount of blood ingested over time remained stable (46, 88). A strain infected by *Wolbachia w*MelPop had decreased blood-feeding success, i.e., increased the number of attempted bites and reduced the blood meal size. Furthermore, a behavior termed as “bendy proboscis” was observed in *w*MelPop-infected mosquitoes after aging, highlighting potential negative effects of aging on mosquito traits (88). By analyzing the number of eggs produced per microliter of ingested blood (in a ratio expressed as eggs/μg), mosquitoes with either *w*Mel or *w*AlbB exhibited reduced egg production compared with *Wolbachia*-uninfected mosquitoes, with a strong effect for *w*Mel and weak effect for *w*AlbB particularly at 3 weeks. Taking the fecundity and blood meal data together, the loss in egg production in older *Wolbachia* mosquitoes seems to be mostly due to an increase in blood meal size rather than a decrease in fecundity, since the number of eggs laid was not affected by mosquito age. One important limitation of our study is that aging and senescence are continuous variables and our aging observations were based on only two points of this continuous distribution, when *Ae. aegypti* females were 7–8 or 21–23 days old. One additional limitation that must be addressed refers to the reduced number of replicates adopted in this experiment. We used eggs derived from two independent cages of each colony. Increasing the number of replicates allows increasing confidence and credibility of the results by reducing the chances of false positives, sampling bias, or measurement error. Thus, our data should be viewed with caution and extrapolations to field must be avoided.

A previous study demonstrated that wing shape can quickly change over just a few generations in *Ae. aegypti*, suggesting microevolutionary adaption (53). Changes in wing shape can be influenced by various environmental and genetic factors (89). For instance, Jaramillo et al. (90) founded that the wing shape of female *Ae. aegypti* was correlated with insecticide resistance levels, which was in turn associated with reduced fecundity and survival. In our study, *Wolbachia* infections had little influence on wing shape variation. In addition, we observed no influence on morphological diversity, i.e., Procrustes variance within the strains. Thus, the wing shape analyses did not indicate a difference in phenotypic variation due to the *Wolbachia* infection. The extent of *Wolbachia*-mediated virus blocking is heterogeneous (91, 92) and depends on factors such as virus serotype, host genetic background, and rearing conditions and the method of infection. Thus, methodological differences between studies may produce different outcomes (27, 55, 93–95). Virus blocking has been positively linked to *Wolbachia* density, with a higher blocking phenotype being verified in specimens with a high density of this bacterium (96). Therefore, a critical trait for the long-term applicability and stability of *Wolbachia* in mitigating arbovirus transmission is its density under natural conditions, especially under fluctuating temperatures (77, 94, 97, 98). Recent data regarding the effects of *Wolbachia* in reducing DENV and CHIKV transmission in Rio de Janeiro revealed a strong seasonal effect on *Wolbachia* introgression in a native population, with a lower frequency of *Wolbachia*-infected mosquitoes during the warmer months (74). However, no information was available for the *Wolbachia* density over the study period. Field data gathered in Cairns, Australia, show the detrimental effects of high temperatures on the stability of *Wolbachia* and its *Ae. aegypti* interaction, affecting maternal transmission, cytoplasmic incompatibility, and likely the potential of *Wolbachia* to mitigate arbovirus transmission (94). Thus, the mosquito-*Wolbachia* density association under a gradient of temperature regimes needs to be carefully investigated for both strains to understand the likely long-term stability of *Wolbachia* as a disease control tool.

Path analysis has been used in ecological studies to describe a myriad of relationships among traits by estimating the reciprocal magnitude of direct and indirect effects (path coefficients) on the response variable (35, 66, 99–101). By combining path analysis and model selection approaches, we explored multiple direct and indirect paths that connect variables involved in blood ingestion and mosquito fecundity for *Ae. aegypti* females transinfected with either *w*Mel or *w*AlbB and uninfected individuals. The best model for the *Wolbachia*-uninfected strain was the full model, whereas reduced models presented better fit for both *Wolbachia*-infected strains. The best model selected for each strain, additionally to resulting in a lower AIC, also provided the best fit according to conventional cutoffs for the model fit indices, enhancing our confidence to interpret model parameter estimates (102). Overall, the relationships revealed by the path analysis were in accordance with generalized linear models. The most intriguing conclusion is that the presence of *Wolbachia* promotes a reshaping of trait pathways regardless of the strain. Noteworthy was the fact the blood meal size, wing length, the number of eggs have no effect on the *Wolbachia* density, a trend observable for both the *w*Mel and *w*AlbB strains. *Wolbachia* is known to affect the reproductive traits of their hosts (31), leading to several physiological and behavioral changes in mosquito biology. Considering both *w*Mel and *w*AlbB strains have been released in several dengue endemic areas of the globe and the well-known fitness costs associated with *Wolbachia* in terms of mosquito fecundity and egg fertility (37, 75), other traits involved in the *Ae. aegypti*-*Wolbachia* interaction could be investigated to better comprehend invasion patterns in endemic settings. For example, it remains unknown to what extension the loss observed in egg fertility and changes in the direct and indirect pathways between fitness traits as reported here through path diagrams can affect the long-term stability of invasions. Determining a path network that includes the infection with an arbovirus like DENV will add new insights into this symbiotic interaction.

In conclusion, our study provides insights into the effects of *Wolbachia* strains *w*Mel and *w*AlbB on various life history traits of *Ae. aegypti* mosquitoes with a controlled genetic background. We observed differential effects on fecundity, blood meal size, and wing shape, which may have implications for the ease with which *Wolbachia* invasion happens in endemic settings (26, 78). For example, a reduction in fecundity of *w*Mel-infected *Ae. aegypti* and a lower production of eggs with a set amount of ingested blood relative to uninfected individuals could slow invasion into native populations. Furthermore, due to egg hatching issues, the native *Wolbachia*-free population can produce a larger egg bank in dry months that synchronously hatch when wet summer conditions start, whereas the eggs from *Wolbachia*-infected eggs (particularly those infected by *w*AlbB) can lose their viability a few weeks after being laid in natural breeding sites (103–105). The interaction between *Wolbachia* and *Ae. aegypti* is complex and influenced by multiple factors, including environmental conditions and mosquito age. To enhance the long-term stability of *Wolbachia* as a tool for mitigating arbovirus transmission, further research is needed to explore additional traits and the influence of virus infections on *Wolbachia*-host interactions. Understanding the intricacies of these interactions will undoubtedly aid in the design and implementation of effective strategies for controlling vector-borne diseases.

## SUPPLEMENTAL MATERIAL

Supplemental material spectrum.00128-24-s0001.pdf Figures S1 and S2.

ASM does not own the copyrights to Supplemental Material that may be linked to, or accessed through, an article. The authors have granted ASM a non-exclusive, world-wide license to publish the Supplemental Material files. Please contact the corresponding author directly for reuse.
