# Supplementary material for: Wolbachia strains wMel and wAlbB differentially affect Aedes aegypti traits related to fecundity
Source: Microbiol Spectr. 2024 Mar 14;12(4):e00128-24. doi: 10.1128/spectrum.00128-24 (PMC10986601; doi:10.1128/spectrum.00128-24)

Supplementary Figure 1: Principal component analysis of the dataset variation. The first two axes explained 56.4% of total variation. Each point corresponds to an adult *Ae. aegypti* female, colored according to mosquito strain: green = *Wolbachia*-uninfected; red = *w*AlbB; blue = *w*Mel.

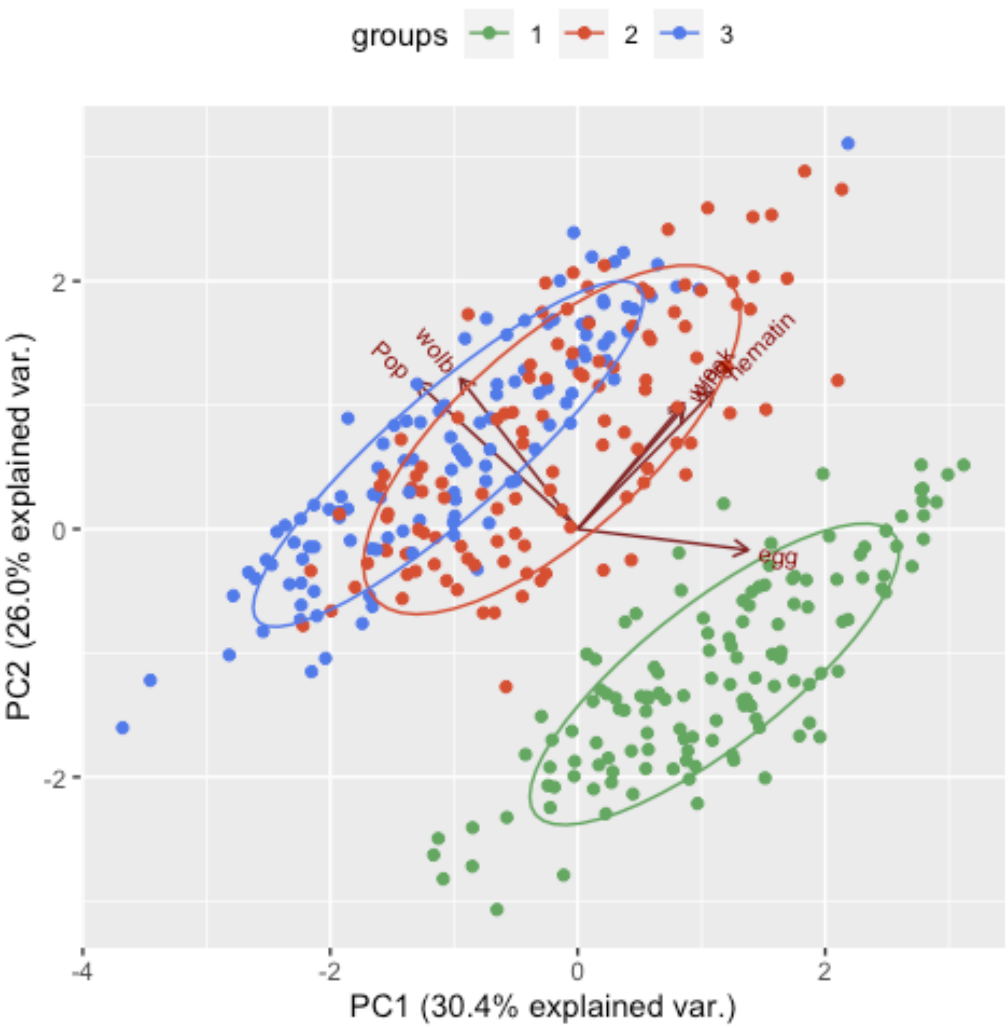

Supplementary Figure 2: Full model path diagrams for: (A) *Wolbachia*-uninfected field population, (B) *w*Mel, and (C) *w*AlbB. Numbers over the arrows are the path coefficients and their statistical significance is indicated as follows: * P < 0.05, ** P < 0.01.

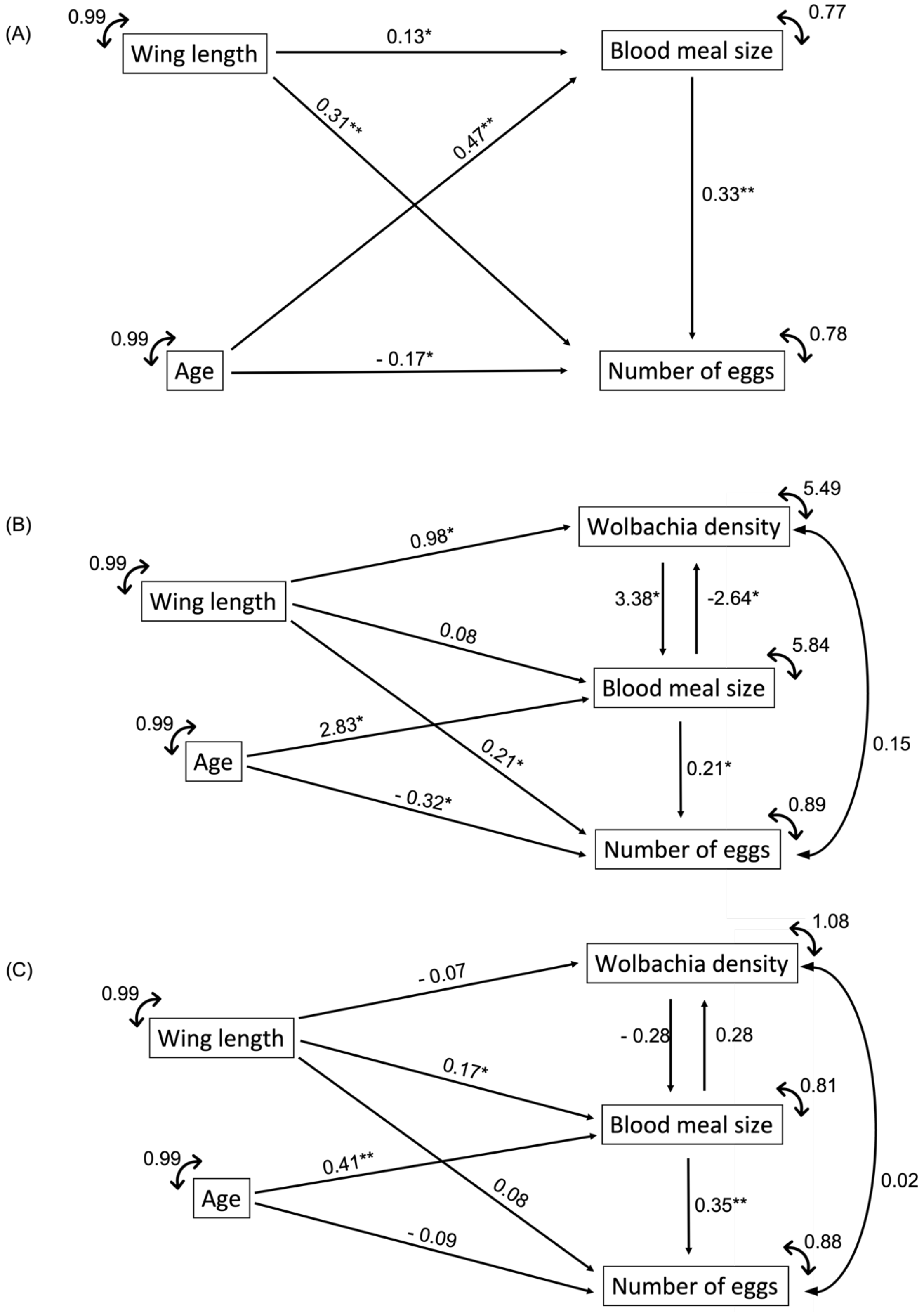

Supplement: Supplemental material — Figures S1 and S2. [file spectrum.00128-24-s0001.pdf]
